# Serglycin induces osteoclastogenesis and promotes tumor growth in giant cell tumor of bone

**DOI:** 10.1038/s41419-021-04161-1

**Published:** 2021-09-23

**Authors:** Yunfei He, Dongdong Cheng, Cheng Lian, Yingjie Liu, Wenqian Luo, Yuan Wang, Chengxin Ma, Qiuyao Wu, Pu Tian, Dasa He, Zhenchang Jia, Xianzhe Lv, Xue Zhang, Zhen Pan, Jinxi Lu, Yansen Xiao, Peiyuan Zhang, Yajun Liang, Qingcheng Yang, Guohong Hu

**Affiliations:** 1 CAS Key Laboratory of Tissue Microenvironment and Tumor, Shanghai Institute of Nutrition and Health, University of Chinese Academy of Sciences, Chinese Academy of Sciences, Shanghai, China; 2 Department of Orthopedics, Shanghai Jiao Tong University Affiliated Sixth People’s Hospital, Shanghai, China; 3 Department of General Surgery, Xinzhou District People’s Hospital, Wuhan, China

**Keywords:** Bone cancer, Cancer microenvironment, Mechanisms of disease

## Abstract

Giant cell tumor of bone (GCTB) is an aggressive osteolytic bone tumor characterized by the within-tumor presence of osteoclast-like multinucleated giant cells (MGCs), which are induced by the neoplastic stromal cells and lead to extensive bone destruction. However, the underlying mechanism of the pathological process of osteoclastogenesis in GCTB is poorly understood. Here we show that the proteoglycan Serglycin (SRGN) secreted by neoplastic stromal cells plays a crucial role in the formation of MGCs and tumorigenesis in GCTB. Upregulated SRGN expression and secretion are observed in GCTB tumor cells and patients. Stromal-derived SRGN promotes osteoclast differentiation from monocytes. *SRGN* knockdown in stromal cells inhibits tumor growth and bone destruction in a patient-derived orthotopic xenograft model of mice. Mechanistically SRGN interacts with CD44 on the cell surface of monocytes and thus activates focal adhesion kinase (FAK), leading to osteoclast differentiation. Importantly, blocking CD44 with a neutralizing antibody reduces the number of MGCs and suppresses tumorigenesis in vivo. Overall, our data reveal a mechanism of MGC induction in GCTB and support CD44-targeting approaches for GCTB treatment.

## Introduction

Giant cell tumor of bone (GCTB) is a common type of primary bone tumor and usually occurs at the metaphysis of the long bones of the limbs, including the distal femur, proximal femur and proximal tibia [1]. Although GCTB is generally considered as a benign tumor and rarely metastasizes, it is locally aggressive and often causes severe bone destruction [2, 3]. There are three main types of cells in GCTB tumor tissues, namely spindle-shaped stromal cells, multinucleated giant cells (MGCs) and monocytes. MGCs are highly similar in both morphology and function to osteoclasts and are considered as the main cause of bone damage by GTCB, while the stromal cells are the neoplastic component in the tumor [4–6]. Current studies show that the neoplastic cells of GCTB are originated from osteoblast-like mesenchymal precursor cells [7, 8] and often harbor the highly specific histone 3.3 G34W (H3.3^G34W^) mutation [9]. In addition, they are known to induce the formation of MGCs from the mononuclear precursors of osteoclasts [10–12]. However, the pathological process of GCTB is poorly studied. In particular, how the stromal cells drive osteoclastogenesis from monocytes is incompletely understood.

Current available treatment options of GCTB are limited. Surgery is the primary treatment, but 27−65% of patients would suffer from recurrence or metastasis after surgery [13]. In addition to surgery, the osteoclast inhibitors bisphosphonates and the anti-RANKL antibody Denosumab are also used in the treatment of GCTB [14, 15]. However, these two drugs have a series of adverse effects. Bisphosphonates usually cause acid reflux and low-grade fever [16, 17], while Denosumab could cause hypocalcemia and hypophosphatemia in patients [18]. Furthermore, GCTB may recur when these drugs are withdrawn [19, 20]. Therefore, there is an urgent need for more effective treatments for GCTB. Better understanding of the pathological interaction among the cell components of GCTB would help find new therapeutic approaches.

SRGN is a low molecular weight glycoprotein first discovered as a secretory product of a rat yolk sac tumor [21]. The core protein is 17.6 kDa in size and contains a 16-amino acid serine/glycine repeat region to which glycosaminoglycan chains are attached [22–24]. SRGN has been extensively studied in the immune system, where it is expressed and essential to the functions of mast cells, cytotoxic T-lymphocytes, macrophages and neutrophils [25–28]. In recent years, studies have shown that SRGN also play important roles in cancer. It is considered as a biomarker of acute myeloid leukemia [29]. In multiple myeloma, high expression of SRGN inhibits the complement activity and helps tumor cells to escape from immune surveillance [30]. In addition, SRGN also regulates the migration and metastasis of breast cancer and lung cancer [31, 32]. However, the roles of SRGN in GCTB or osteoclastogenesis are unclear. Here, we report that stromal-secreted SRGN interacts with CD44 of monocytes to promote MGC formation in GCTB.

## Results

### SRGN expression and secretion are upregulated in GCTB

**Fig. 1:**
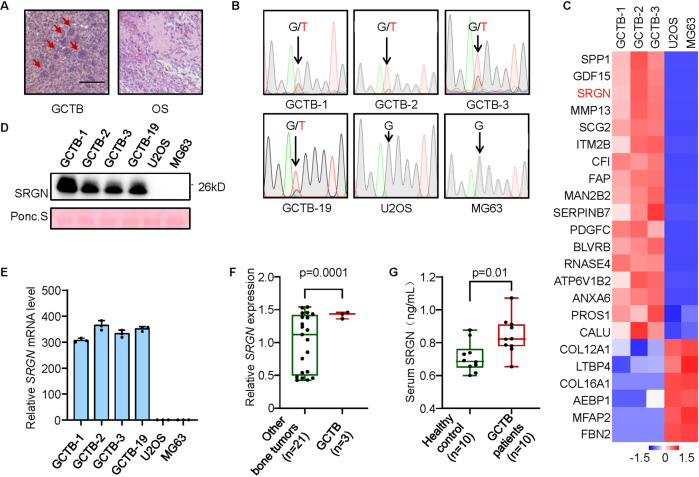
Expression and secretion of SRGN are upregulated in GCTB. **A** Hematoxylin and eosin (H&E) staining of GCTB and osteosarcoma (OS) tissues. Arrows point to osteoclast-like MGCs in GCTB. **B** Genomic sequencing of H3.3 mutations in GCTB primary cell lines (GCTB-1, GCTB-2, GCTB-3 and GCTB-19) and OS cell lines (U2OS and MG63). **C** Heatmap of mass-spectrum secretomic analysis of GCTB and OS cells. **D**, **E** SRGN secretion (**D**) and mRNA levels (**E**) in GCTB and OS cells. **F** SRGN expression in various bone tumor cell lines in the Cancer Cell Line Encyclopedia database. **G** Serological SRGN levels of GCTB patients and healthy people. Scale bar, 100 μm. *P* values were obtained by two-tailed unpaired *t* test (**F**, **G**). Box plots display values of minimum, first quartile, median, third quartile, and maximum. Bar graphs are shown as mean ± s.d.

To study GCTB, we established a series of primary stromal cell lines from clinical GCTB tumors (Supplementary Fig. S1A). These tumors and primary lines display characteristic MGC presence (Fig. 1A). Most of the GCTB tumors harbored the H3.3^G34W^ mutation (Fig. 1B). We performed mass-spectrum secretomic profiling of GCTB primary cells with two osteosarcoma cell lines as the control. The analysis identified 23 differentially secreted proteins, among which secreted phosphoprotein 1 (SPP1), growth differentiation factor 15 (GDF15) and SRGN ranked at the top of upregulated proteins in GCTB (Fig. 1C). SPP1, also known as OPN, is a well-known factor with critical roles in osteoclastogenesis and cancer-related osteolysis [33]. Although GDF15 was previously reported by Hinoi et al. [34] to regulate hypoxia-driven osteoclastic differentiation, we found that knockdown of *GDF15* in GCTB stromal cells resulted in no obvious changes in the ability of the cells to induce osteoclast differentiation from primary bone marrow cells (Supplementary Fig. S1B–D), indicating that GDF15 might not play a major role in the MGC formation of GCTB. Therefore, we focused on SRGN, which has not been studied in GCTB or bone remodeling, in our analyses.

We first verified the upregulation of SRGN in GCTB. Consistent with the mass-spectrum analysis, quantitative PCR (qPCR) and Western blotting assays showed that the mRNA expression and protein secretion of SRGN were much higher in GCTB stromal cells than in osteosarcoma cells (Fig. 1D, E). The expression of *SRGN* was also significantly upregulated in GCTB cell lines than in other bone tumor cell lines, including chondrosarcoma, Ewing sarcoma and osteosarcoma, in the Cancer Cell Line Encyclopedia database [35] (Fig. 1F). SRGN was also mildly expressed in bone-metastatic breast cancer cell lines SCP2 and 1833 [36], but not in normal bone stroma cells including mesenchymal stem cells, osteoblasts and osteoclasts (Supplementary Fig. S1E). Upregulation of SRGN in GCTB was likely independent of H3.3^G34W^ mutation, as its high expression in a GCTB tumor without H3.3^G34W^ mutation, GCTB-4, was also observed (Supplementary Fig. S1F). We further analyzed the serum samples of GCTB patients and found that the serological SRGN levels were significantly higher in GCTB patients than in healthy people (Fig. 1G), further confirming the enhanced secretion of SRGN by GCTB cells.

### SRGN promotes osteoclastic differentiation in vitro

**Fig. 2:**
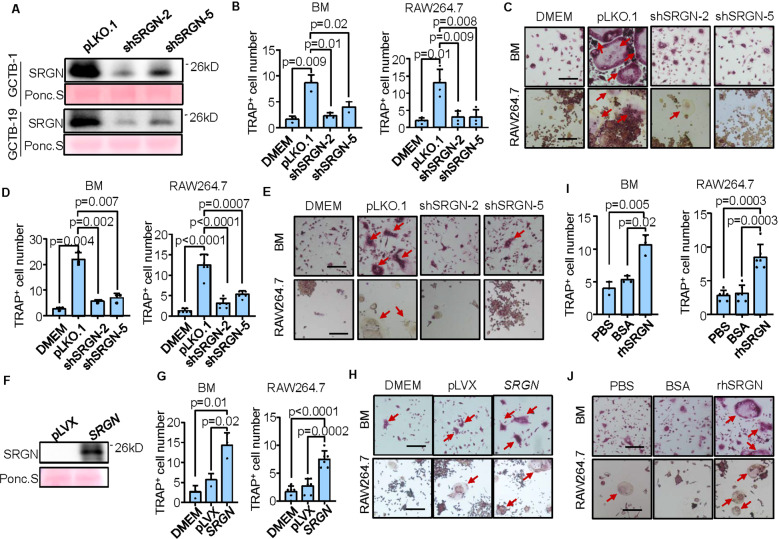
SRN promotes osteoclastogenesis in vitro. **A** Western blotting analysis to validate *SRGN* knockdown in GCTB-1 and GCTB-19. **B**, **C** Osteoclast quantification (**B**) and representative images (**C**) in mouse primary bone marrow (BM) and RAW264.7 cultured in DMEM medium or conditioned media (CM) of GCTB-1 with or without *SRGN* knockdown in osteoclastogenesis assays. **D**, **E** Osteoclast quantification (**D**) and representative images (**E**) in mouse primary bone marrow and RAW264.7 cultured in DMEM medium or CM of GCTB-19 with or without *SRGN* knockdown in osteoclastogenesis assays. **F** Western blotting analysis to validate *SRGN* overexpression in hFOB1.19. **G**, **H** Osteoclast quantification (**G**) and representative images (**H**) in mouse primary bone marrow and RAW264.7 cultured in DMEM medium or CM of hFOB1.19 with or without *SRGN* overexpression in osteoclastogenesis assays. **I**, **J** Osteoclast quantification (**I**) and representative images (**J**) in mouse primary bone marrow and RAW264.7 treated with or without human recombinant SRGN protein (25 ng/mL) in osteoclastogenesis assays. Scale bar, 100 μm. Arrows point to giant osteoclast cells (**C**, **E**, **H**, **J**). *P* values were obtained by two-tailed unpaired *t* test (**B**, **D**, **G**, **I**). Bar graphs are shown as mean ± s.d.

To study the function of SRGN in GCTB, we knocked down *SRGN* in a GCTB primary cell line GCTB-1 (Supplementary Fig. S2A). The conditioned medium from GCTB-1 was used to induce osteoclastic differentiation of mouse primary bone marrow cells and the RAW264.7 monocyte cells. *SRGN* knockdown inhibited the secretion of SRGN into conditioned medium (Fig. 2A) and significantly decreased the number of mature osteoclasts differentiated from bone marrow and RAW264.7 when cultured in GCTB-1 medium (Fig. 2B, C). Similar effects were observed when *SRGN* was knocked down in another GCTB stromal cell line GCTB-19 (Supplementary Fig. S2B and Fig. 2A, D, E). Since the neoplastic stromal cells of GCTB were originated from osteoblast-like mesenchymal precursors, we tested whether *SRGN* overexpression in the human osteoblast precursor cell line hFOB1.19 was sufficient to enhance osteoclastogenesis. *SRGN* overexpression in hFOB1.19 elevated SRGN secretion (Fig. 2F) and concordantly, promoted osteoclastogenesis from mouse bone marrow cells and RAW264.7 when they were incubated with hFOB1.19 conditioned medium (Fig. 2G, H). In addition, when bone marrow cells and RAW264.7 cells were treated with recombinant SRGN protein, osteoclastogenesis was significantly enhanced (Fig. 2I, J). Notably, the above osteoclastogenesis assays were preformed with RANKL, a fundamental cytokine for osteoclast differentiation which is known to be also upregulated in GCTB [37–39]. Further analyses showed that the promoting effect of SRGN on osteoclastogenesis was weaker than that of RANKL. When RANKL was removed from the osteoclastogenesis assays, the effect of recombinant SRGN protein on osteoclastogenesis also became weaker (Supplementary Fig. S2C, D). These data suggested that stromal-derived extracellular SRGN contributed to the formation of osteoclast-like MGCs in GCTB, although its effect seemed not as prominent as that of RANKL.

In addition, we observed that GCTB-19 cells could induce RAW264.7 secretion of the pro-tumor cytokine IL-6, and the conditioned medium of GCTB-19-induced RAW264.7 in turn enhanced the proliferation of GCTB-19 cells. When *SRGN* was knocked down in GCTB-19, IL-6 secretion by RAW264.7 after GCTB-19 induction was suppressed, and the promoting effect of RAW264.7 medium on GCTB-19 proliferation was also significantly reduced (Supplementary Fig. S2E, F), corroborating an effect of SRGN-induced osteoclastogenesis to promote GCTB growth.

### SRGN is required for MGC formation and GCTB tumorigenesis in vivo

**Fig. 3:**
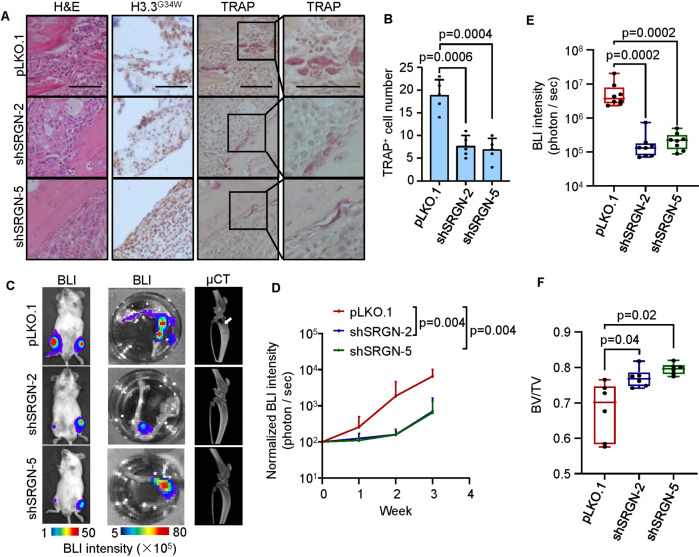
SRGN inhibition suppresses MGC formation and tumor growth of GCTB in vivo. **A** H&E, IHC, and TRAP staining of bone sections after intratibial injection of GCTB-19 cells with *SRGN* knockdown in NOD/SCID mice. **B** Quantification of TRAP-positive cells in bone sections. **C** Representative images of bioluminescent imaging (BLI) analyses of the whole bodies and hind limbs of the mice, and micro-CT analyses for bone destruction of hind limbs. Arrows point to osteolytic areas in the legs. **D** Weekly BLI quantitation of tumor burden of the mice (*n* = 6 mice per group). **E** Ex vivo BLI quantitation of tumor burden in hind limbs. **F** Micro-CT quantification of relative bone volumes of the mice. BV/TV bone volume/total volume. Scale bar, 100 μm. *P* values were obtained by Mann−Whitney *U* test (**D**, **E**) and two-tailed unpaired *t* test (**A**, **F**). Box plots display values of minimum, first quartile, median, third quartile, and maximum. Bar graphs are shown as mean ± s.d.

Then we further tested the in vivo function of SRGN in GCTB. As GCTB cell lines for xenograft analysis has been previously lacking, we screened the GCTB primary stromal cell lines established by us by intratibial injection of them into immunodeficient NOD/SCID mice. One of the cell lines GCTB-19, which also harbored the H3.3^G34W^ mutation, resulted in osteolytic tumors in the bone (Fig. 3A). More importantly, tartrate-resistant acid phosphatase (TRAP) staining of bone lesions revealed the presence of TRAP^+^ multinucleated osteoclasts within the tumor areas in addition to the tumor-bone interface (Fig. 3A). This indicated a characteristic feature of GCTB and was different to the bone metastases caused by carcinoma cells, such as breast cancer cells, where osteoclasts are usually found along the tumor-bone interface. The tumors were also positive for H3.3^G34W^ mutation (Fig. 3A). Interestingly, *SRGN* knockdown in GCTB-19 led to much less TRAP^+^ osteoclasts in the xenograft tumors (Fig. 3A, B). Notably, immunostaining analysis showed that SRGN was mainly expressed in the tumor area of GCTB, but not in para-tumor stroma (Supplementary Fig. S3). After *SRGN* knockdown, osteoclasts within the tumors were no longer observed (Fig. 3A). We also labeled the GCTB-19 cells with the firefly luciferase and quantitated the xenograft tumor growth in mice by bioluminescent imaging (BLI). Weekly BLI analysis showed that *SRGN* knockdown markedly reduced GCTB tumor burden of mice (Fig. 3C, D). Ex vivo analyses of the hind limbs of mice also revealed a nearly 30 times reduction of tumor growth after *SRGN* knockdown by the third week after intratibial injection (Fig. 3C, E). In addition, microCT analysis showed the control GCTB-19 tumors resulted in severe bone destruction, while *SRGN* silencing led to much milder bone damages and recovered the bone volumes (Fig. 3C, F). Collectively, these data demonstrated a role of SRGN in MGC formation and GCTB tumorigenesis.

### SRGN promotes osteoclastic differentiation through CD44

**Fig. 4:**
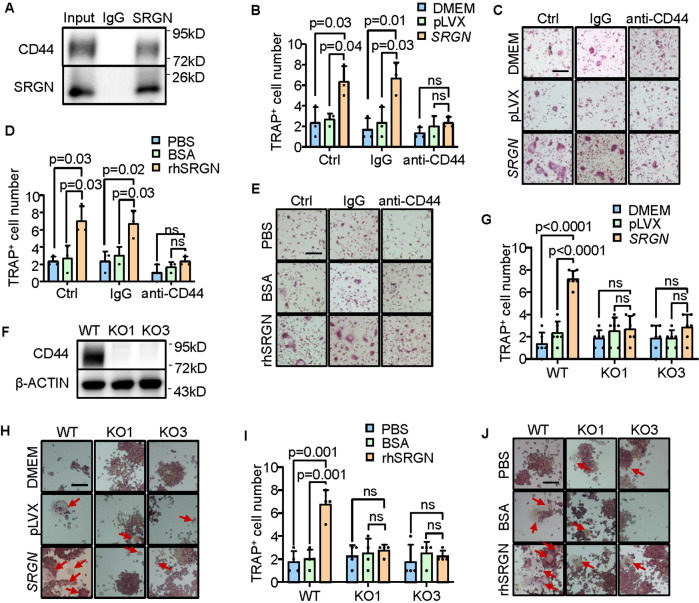
SRGN functions through its receptor CD44. **A** Co-immunoprecipitation of SRGN and CD44. RAW264.7 and GCTB-19 cells were cocultured in a ratio of 5:1 for 48 h. Cell lysates were immunoprecipitated with an anti-SRGN antibody followed by immunoblotting with anti-CD44 and anti-SRGN antibodies. **B**, **C** Osteoclast quantification (**B**) and representative images (**C**) of mouse primary bone marrow with the treatment of hFOB1.19 CM and the CD44 neutralizing antibody (10 ng/mL) in the osteoclastogenesis assay. **D**, **E** Osteoclast quantification (**D**) and representative images (**E**) of mouse primary bone marrow with the treatment of human recombinant SRGN protein (rhSRGN) and the CD44 neutralizing antibody (10 ng/mL). **F** Validation of *CD44* knockout (KO) in RAW264.7 cells. **G**−**J** Osteoclastogenesis assay of RAW264.7 *CD44*-knockout cells with treatment of hFOB1.19 CM (**G**, **H**) or human recombinant SRGN protein (**I**, **J**). Shown are the numbers of giant mature osteoclasts (**G**, **I**) and representative images (**H**, **J**). Scale bar, 100 μm. *P* values were obtained by two-tailed unpaired *t* test (**B**, **D**, **G**, **I**). Bar graphs are shown as mean ± s.d.

Since SRGN is a secreted protein, we hypothesized that it might regulate monocyte differentiation by binding to a surface protein of monocytes. Thus, we performed immunoprecipitation of cocultured RAW264.7 and GCTB-19 cells with an SRGN antibody, followed by mass-spectrum analysis of the precipitated proteins. Among the nine identified proteins (Supplementary Fig. S4A), CD44 was previously reported as a receptor of SRGN in T cells [40]. We confirmed the binding of SRGN to CD44 in RAW264.7 by co-immunoprecipitation (co-IP) assay (Fig. 4A). CD44 is expressed in different isoforms. Reciprocal co-IP assays further showed that SRGN bound to both the standard isoform (CD44s) and the variant isoform (CD44v3-v10) of CD44 (Supplementary Fig. S4B–E).

Therefore, we tested whether CD44 played a role in SRGN-induced osteoclastic differentiation. Primary mouse bone marrow cells were incubated in conditioned medium from *SRGN*-overexpressing hFOB1.19 cells, together with a CD44 neutralizing antibody or IgG control, followed by osteoclastogenesis analysis. It was shown that *SRGN* overexpression promoted the generation of mature osteoclasts, while CD44 inhibition suppressed osteoclastogenesis and abolished the effect of *SRGN* (Fig. 4B, C). The similar phenomenon was observed when the SRGN recombinant protein was used to induce osteoclast differentiation. With the treatment of CD44 neutralization, recombinant SRGN was no longer able to promote osteoclastogenesis (Fig. 4D, E). The assays were also repeated in RAW264.7 cells and consistent results were observed (Supplementary Fig. S4F−I). In addition, we used the CRISPR-Cas9 system to knock out *CD44* in RAW264.7 (Fig. 4F). After *CD44* knockout, either conditioned medium from *SRGN*-overexpressing hFOB1.19 cells or the SRGN recombinant protein could no longer promote osteoclastic differentiation of RAW264.7 cells (Fig. 4G−J). All together, these results indicated that SRGN regulates osteoclastic differentiation of monocytes through CD44.

### SRGN activates focal adhesion kinase (FAK) through CD44

**Fig. 5:**
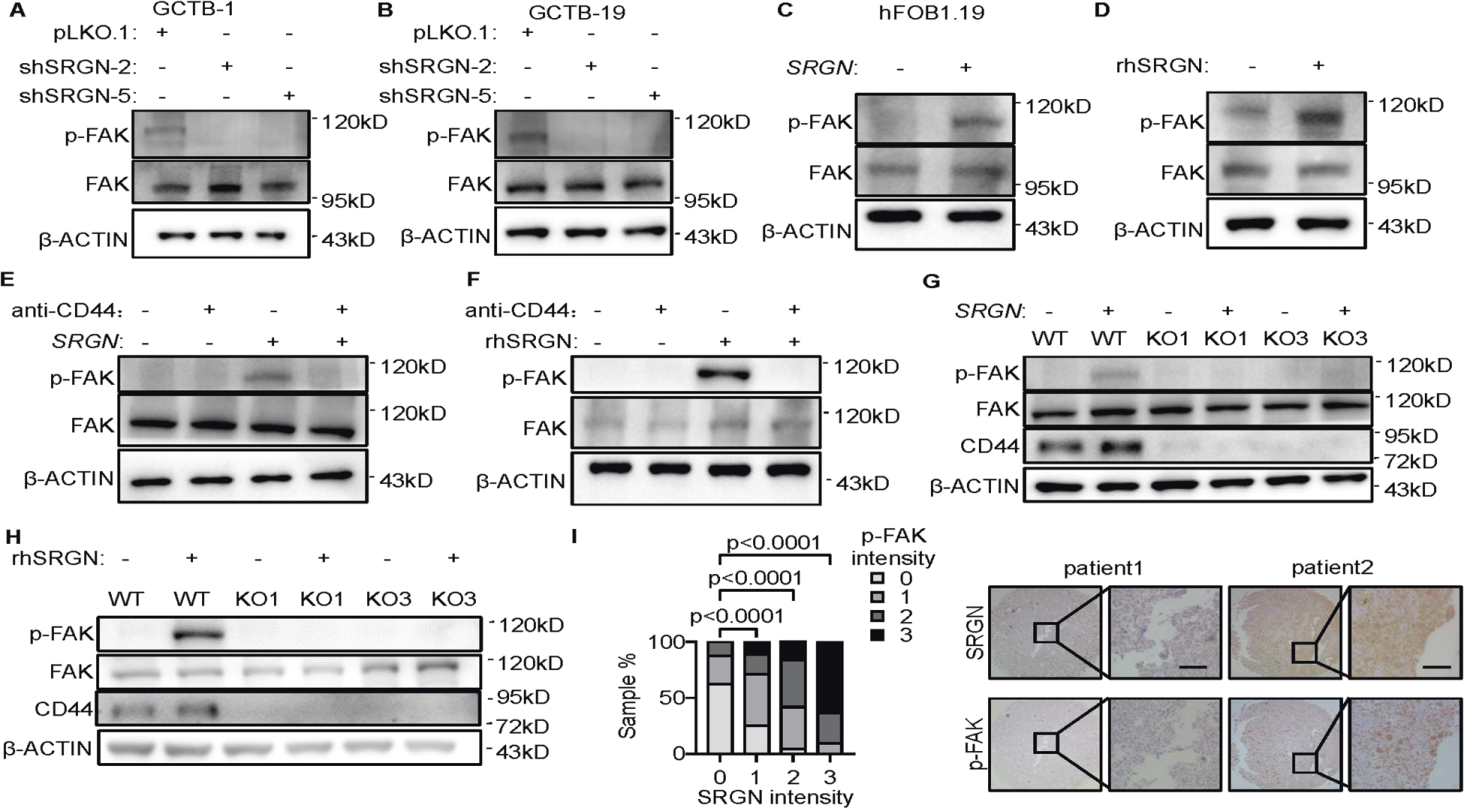
SRGN activates focal adhesion kinase through CD44. **A**−**D** Western blot analysis of phosphorylated FAK protein level in RAW264.7 cells after treatment with CM from GCTB-1 (**A**) or GCTB-19 (**B**) with *SRGN* knockdown, CM from hFOB1.19 with *SRGN* overexpression (**C**), or human recombinant SRGN protein (**D**). **E** Western blotting analysis of phosphorylated FAK protein level in RAW264.7 cells after treatment with CM from hFOB1.19 with *SRGN* overexpression and the CD44 neutralizing antibody. **F** Western blotting analysis of phosphorylated FAK in RAW264.7 cells after treatment with human recombinant SRGN protein and the CD44 neutralizing antibody. **G**, **H** Western blotting analysis of phosphorylated FAK in RAW264.7 *CD44*-knockout cells after treatment with CM from hFOB1.19 with *SRGN* overexpression (**G**) and human recombinant SRGN protein (**H**). **I** FAK phosphorylation levels in human GCTB samples with different levels of SRGN expression. Protein expression was scored as 0 (negative), 1 (weak), 2 (moderate) and 3 (strong) by immunohistochemistry staining (*n* = 71 patients). Scale bar, 100 μm. *P* values were obtained by chi-squared test (**I**).

Next, we sought to delineate the downstream mechanism of CD44 when bound with SRGN. It has been reported that focal adhesion kinase (FAK) is one of the downstream molecules that could be activated by CD44 signaling [41] and, importantly, FAK is well known to be crucial for the function of osteoclasts, as well as MGCs in GCTB, by regulating adhesion structures and cytokine signaling of osteoclasts [42–44]. Hence, we analyzed whether SRGN could regulate FAK. Treating RAW264.7 cells with conditioned media of GCTB-1 and GCTB-19 led to FAK phosphorylation in RAW264.7, while *SRGN* knockdown in these GCTB cells distinctly reduced FAK phosphorylation (Fig. 5A, B). Reciprocally, both conditioned medium from *SRGN*-overexpressing hFOB1.19 cells and SRGN recombinant protein significantly increased the phosphorylation of FAK in RAW264.7 cells (Fig. 5C, D). In contrast, when CD44 of RAW264.7 was inhibited by the neutralizing antibody, neither conditioned medium from *SRGN*-overexpressing hFOB1.19 cells nor SRGN recombinant protein could activate FAK (Fig. 5E, F). We also repeated these experiments in primary mouse bone marrow cells and observed the same phenomena (Supplementary Fig. S5A−F). In addition, *CD44* knockout in RAW264.7 also abolished the effect of *SRGN*-overexpressing hFOB1.19 conditioned medium and SRGN recombinant protein to activate FAK of the monocytes (Fig. 5G, H). In addition, the regulation of FAK signaling by SRGN was independent of RANKL (Supplementary Fig. S5G). We further used an FAK inhibitor, Defactinib, to treat the monocytes. With the inhibitor, the conditioned media from GCTB-1 or GCTB-19 cells could no longer promote osteoclastogenesis (Supplementary Fig. S5H, I). These results showed that SRGN binds to CD44 on the surface of monocytes to activate the downstream FAK signaling pathway for osteoclastic differentiation.

Furthermore, we analyzed the expression of SRGN and FAK phosphorylation in clinical GCTB tissues by immunostaining of a human GCTB tissue microarray. The analysis revealed a significant positive correlation between SRGN expression and FAK activation in human tumor samples (Fig. 5I), thus corroborating the link of SRGN to FAK signaling in GCTB.

### Targeting CD44 with the neutralizing antibody suppresses GCTB tumorigenesis in mice

**Fig. 6:**
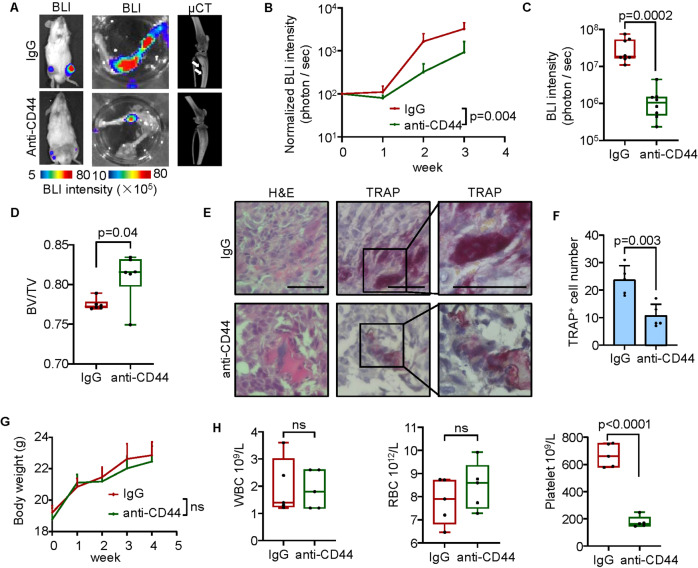
CD44 blocking with the neutralizing antibody suppresses GCTB in vivo. **A** Representative images of BLI analyses of the whole bodies and hind limbs, and micro-CT analyses for bone destruction of hind limbs of the mice with or without anti-CD44 treatment after intratibial injection of GCTB-19 cells in NOD/SCID mice. Arrows point to osteolytic areas in the legs. **B** Weekly BLI quantification of whole-body tumor burden of the mice (*n* = 6 mice per group). **C** Ex vivo BLI quantification of tumor burden in hind limbs. **D** Micro-CT quantification of relative bone volumes of the mice. **E** H&E and TRAP staining of bone sections. **F** Quantification of TRAP-positive cells in bone sections. **G** Body weights of healthy mice injected with the CD44 neutralizing antibody or control IgG. **H** Blood components of healthy mice injected with the CD44 neutralizing antibody or control IgG. WBC white blood cell, RBC red blood cell. Scale bar, 100 μm. *P* values were obtained by Mann−Whitney *U* test (**B**, **C**) and two-tailed unpaired *t* test (**D**, **F**, **G**, **H**); ns not significant. Box plots display values of minimum, first quartile, median, third quartile, and maximum. Bar graphs are shown as mean ± s.d.

Thus far we had affirmed the role of SRGN-CD44 signaling in osteoclastogenesis and tumorigenesis of GCTB, and therefore we investigated whether the SRGN-CD44 axis could be targeted for GCTB treatment. The GCTB-19 cells were inoculated into the tibia of NOD/SCID mice, followed by intraperitoneal injection of the CD44 neutralizing antibody a week later. Each animal was treated with 100 μg CD44 neutralizing antibody or control IgG every other day. BLI signals showed that the tumor burden of the mice was greatly reduced after treatment with the antibody (Fig. 6A, B). Two weeks after the treatment, the tumor signals in hind limbs were reduced by over ten times, as shown by ex vivo analyses of the limbs (Fig. 6A, C). Consistently, CD44 blocking also salvaged the mice from bone damage by GCTB (Fig. 6A, D). TRAP staining of the bone lesions also revealed a significant reduction in the number of osteoclast-like MGCs after the treatment of CD44 neutralization (Fig. 6E, F). These data showed the effectiveness of CD44 inhibition for GCTB treatment.

Further we evaluated the safety of CD44 targeting by the neutralizing antibody. Healthy mice were treated by intraperitoneal administration of the neutralizing antibody or IgG control in the same dosage of the above experiments testing the antibody effectiveness, 100 μg per mouse every other day, but for up to 4 weeks. The body weights and blood composition of mice were monitored. It was observed that continuous anti-CD44 treatment had no significant effect on body weight (Fig. 6G). Although the treatment led to a drop in the number of platelets in the blood, the numbers of white and red blood cells were almost unchanged (Fig. 6H). Taken together, these results argued for the potential of CD44 targeting as a therapeutic strategy to treat GCTB.

## Discussion

GCTB is a common bone tumor with relatively high recurrence rate, but the pathogenesis and tumor biology of GCTB have been under-investigated. Although it has been shown that GCTB shares regulatory pathways of osteoclastogenesis with other osteolytic primary bone tumors and bone metastases, the unique features of GCTB including the abundant within-tumor osteoclast-like MGCs suggest distinct regulatory mechanisms for GCTB. However, many of previous studies of GCTB were restricted with in vitro analyses due to the lack of suitable GCTB animal models that can recapitulate the main features of the disease. Some studies used in vivo models by subcutaneously injecting the stromal cells into mice [45, 46] or growing tumor tissues on chick chorioallantoic membranes (CAM) [47–49], but these models only assess the growth of tumor cells and cannot produce giant cells or bone lesions. Recently, intratibial inoculation of patient-derived tumor cells into immunodeficient mice was proposed to establish an orthotopic model, offering the possibility to study in situ stromal-osteoclast interaction in GCTB [50–52], but the success rate of this model is low and it had not been used for mechanistic exploration of GCTB osteoclastogenesis. In this study, we established a series of primary cell cultures from GCTB tumors, and among these primary cell lines, further identified GCTB-19 that was capable to form tumors in bone with characteristics of GCTB, including MGCs and bone absorption. With these models, we identified SRGN, which had not been previously implicated in bone diseases, with a critical role in stromal induction of MGCs and bone destruction. We further delineated the downstream pathway in which SRGN binds to its receptor CD44 on the monocyte surface and activates FAK for MGC formation. Importantly, the role of SRGN and the effectiveness of CD44 targeting are validated using the patient-derived orthotopic xenograft model. Thus, our study provides an example to use clinically relevant animal models to identify new regulatory factors and therapeutic targets for GCTB.

Our data also showed the upregulation of SRGN in GCTB in comparison of other bone tumors and healthy control. Importantly, the GCTB patients display a much higher serological SRGN level, implicating a potential value of SRGN for GCTB diagnosis. Currently GCTB is diagnosed mainly by histopathological and radiological evaluation, as well as examination of H3.3^G34W^ mutation [53, 54]. However, some other bone tumors, such as giant cell-rich osteosarcoma [55], may share histopathological and radiological similarity with GCTB. In addition, although H3.3^G34W^ is highly specific to GCTB, a small portion of tumors are still negative for this mutation. Thus, additional markers would be useful to supplement the current diagnostic approaches. However, further studies, including validation in larger clinical cohorts, would be needed to establish SRGN as a histological or serological marker of GCTB.

In addition, our study also provided the evidence to support CD44 targeting to treat GCTB. Treatment of the mice with the CD44 neutralizing antibody significantly suppressed tumor growth and alleviated bone damage, suggesting a possible option for adjuvant therapy of GCTB in addition to currently used bisphosphonates and Denosumab. However, CD44 is expressed in a wide variety of cell types and plays important roles in various physiological and pathological conditions. Therefore, CD44 targeting might be accompanied with some undesirable side effects. Although our preliminary analyses showed that the antibody treatment did not elicit severe effects in healthy animals, a decrease in platelets was observed. This might be reflective to previously studies indicating the roles of CD44 in platelet hemostasis and function [56, 57]. Nevertheless, the safety and efficacy of CD44 blocking for GCTB treatment are to be further investigated. Instead, alternative approaches to target the SRGN-CD44-pFAK signaling could also be considered. For example, FAK inhibitors also demonstrated promising effect to inhibit osteoclastogenesis (Supplementary Fig. S5H, I). Finding SRGN inhibitors to directly target SRGN or SRGN-CD44 interaction could be important to develop new therapeutic approaches. As SRGN is a secreted protein, developing a neutralizing antibody against SRGN is a possible strategy.

## Materials and methods

### Primary GCTB cell culture

The GCTB cells were isolated from tumor samples derived from tumor resections in Shanghai Sixth People’s Hospital. The tissues were mechanically cut into small pieces and digested with 1.5 mg/mL collagenase B for 3 h at 37 °C in Dulbecco’s modified eagle medium (DMEM) containing 4.5 g/L glucose and supplemented with 10% fetal bovine serum (FBS), 100 U/mL penicillin and 100 mg/mL streptomycin. Cells were collected by filtration (100-mm-diameter filter) centrifugation and washed twice in phosphate buffered solution (PBS). The cells were cultured in humidified air with 5% CO_2_ at 37 °C. Culture medium was changed every 2−3 days until approximately 80% confluence. After several successive passages, the culture became homogeneous of spindle-shaped stromal cells, and other cell types were eliminated. These cells were used for subsequent in vitro and in vivo assays.

### Constructs and reagents

Human *SRGN* and *CD44* were constructed into the pLVX-puro and pCDNA3.1 vectors (Clontech), respectively, for overexpression. The annealed sense and antisense shRNA oligonucleotides were cloned into the pLKO.1-puro vector (Addgene) for knockdown of human *SRGN* with the following target sequences: CCAGGACTTGAATCGTATCTT (shSRGN#2), ACATGGATTAGAAGAGGATTT (shSRGN#5). The annealed sense and antisense sgRNA oligonucleotides were cloned into pX458 vector for knockout of murine *Cd44* with the following target sequences: AATGTAACCTGCCGCTACGC (sgCd44#1), GGGAGGTGTTGGACGTGACG (sgCd44#3). The antibodies used for Western blotting, immunoprecipitation and immunohistochemistry were as follows: β-ACTIN (A2228, Sigma), Flag (F1804, Sigma), SRGN (sc-374657, Santa Cruz), CD44 (37259, CST), His (12698, CST), FAK (A11531, Abclonal), phosphor-FAK (AP0302, Abclonal), H3.3^G34W^ (RM263, RevMAb). The CD44 neutralizing antibodies were obtained from Thermo Fisher Scientific (14-0441-82) for in vitro treatment and from Bio X Cell (BE0039) for in vivo treatment. The human SRGN recombinant protein was from Sino Biological (13648-H08H). The murine RANKL recombinant protein (Peprotech, 315-11) and the murine M-CSF recombinant protein (Peprotech, 315-02) were used in this study. The FAK inhibitor Defactinib for in vitro assay (2 μM) was obtained from MedChemExpress (HY-12289).

### Osteoclastogenesis assays

Osteoclastogenesis was conducted with bone marrow harvested from 4- to 7-week-old BALB/c mice or RAW264.7 cell lines. Conditioned medium (CM) from cancer cells was mixed with α-MEM (supplied with 20% FBS, 25 ng/mL RANKL) at a 1:3 ratio for osteoclastic differentiation. Unless stated otherwise, 25 ng/mL RANKL was supplemented in the osteoclastogenesis medium. Various antibodies and recombinant proteins were administrated directly into the CM-α-MEM mixture, as specified for each experiment.

### Western blotting

Cultured cells were rinsed with pre-cooled PBS and lysed by lysis buffer (50 mM Tris-HCl, 150 mM NaCl, 1% Nonidet P-40, 0.5% sodium deoxycholate, 0.1% SDS with phosphatase and protease inhibitors) at 4 °C for 15 min, followed by centrifugation at 10,000 × *g* for 15 min. The supernatants were collected, quantified and denatured for Western blot analysis. For secreted protein, 0.25 mL of trichloroacetic acid was added to 1 mL CM. After incubation on ice for 1 h, the samples were spun at 10,000 × *g* for 30 min and the supernatants were discarded. Pellets were washed twice by spinning at 10,000 × *g* for 5 min in cold acetone and resuspended in SDS loading buffer. The proteins were separated by 10 or 12% SDS-PAGE and transferred onto a cellulose acetate membrane. The membrane was stained with Ponceau S and blocked by 5% nonfat milk or 5% BSA in phosphate-buffered saline with 0.1% Tween 20 for 1 h at room temperature. The membrane was then incubated with a primary antibody overnight and washed, followed by blotting with a secondary antibody conjugated with HRP for 1 h at room temperature. The signals were visualized with chemiluminescent HRP substrate.

### Co-IP and mass-spectrum (MS) analyses

Cell lysates were centrifuged at 10,000 × *g* for 15 min at 4 °C to remove intact cells. The supernatant was either incubated with control immunoglobulin G (IgG) or primary antibody overnight in IP buffer (150 mM NaCl, 20 mM HEPES at pH 7.4, 1% Triton X-100, 12.5 mM β-glycerophosphate, 1.5 mM MgCl_2_, 2 mM ethylenebis (oxyethylenenitrilo) tetraacetic acid (EGTA) with phosphatase and protease inhibitors), followed by incubation with 20 μL of resuspended volume of Protein A/G beads (GE Life Sciences) for 2 h at 4 °C to pull down bound proteins. Beads were centrifuged at 1000 × *g* for 5 min at 4 °C to remove the supernatant, washed four times with the IP buffer and boiled for 20 min at 95 °C. Samples were run on SDS-PAGE gel, followed by Coomassie Brilliant Blue R-250 (Bio-Rad) staining. Afterwards, gel bands were excised, destained, trypsinized and subjected to MS analysis to identify individual proteins using liquid chromatography-MS (Orbitrap Velos Pro mass spectrometer, Thermo Fisher Scientific).

### Mouse experiments

All animal studies were conducted according to the guidelines for the care and use of laboratory animals and were approved by the Institutional Animal Care and Use Committee of Shanghai Institute of Nutrition and Health. The intratibial bone injection was performed in 4- to 5-week-old, male NOD/SCID mice. 1 × 10^7^ GCTB cells were resuspended in 1 mL PBS. Each leg of the mouse was injected with 20 μL cell suspension. BLI data were acquired with an IVIS Spectrum CT system (PerkinElmer). MicroCT data were acquired with a vivaCT80 (Scanco) system. For blood components analysis, blood samples (50 μL) were collected using tubes and immediately diluted by PBS containing 5 mM ethylene diamine tetraacetic acid (EDTA) into 100 μL, and analyzed by an Auto Hematology Analyzer (Mindray, BC-2800 Vet). CD44 neutralizing antibody treatment was performed by intraperitoneal injection 1 week after tumor inoculation. Each mouse was injected with 100 μg antibody (Bio X Cell, NH, USA) every other day. No statistical method was used to predetermine the sample size of animal studies. Mice were randomly grouped with approximately equal body weight between groups. No mice were excluded from analyses except those with unexpected death from non-tumor reason. Investigators were not blinded to allocation during the experiments and outcome assessment.

### TRAP staining

TRAP staining was performed with the tartrate-resistant acid phosphatase kit (Sigma 387A). Osteoclast numbers were assessed as multinucleated TRAP^+^ cells in each field of view.

### Clinical analysis

Fresh human GCTB samples, paraffin-embedded human GCTB tissues for microarray construction, and human serum samples from GCTB patients and healthy individuals were obtained from Shanghai Sixth People’s Hospital with informed consent from all participants and approval from the Hospital’s Research Ethics Committee. SRGN and phosphor-FAK were immunostained and scored as negative (0), weak (1), moderate (2) or strong (3) according to staining intensities. The SRGN ELISA kit (SEC869Hu, USCN) was used to analyze serological levels of SRGN in the serum samples.

### Statistical analyses

Data analyses were performed using GraphPad Prism 8.0 (GraphPad Software, La Jolla, USA). The data presentation and statistical analyses are described in the figure legends. *P* values < 0.05 were considered as statistically significant. The experiments in vitro were repeated independently multiple times with similar results, as indicated in the figure legends.

## Supplementary information

Supplementary Figures

## Data Availability

Raw data in this study are available upon request to the corresponding authors.
