# Correction: Serglycin induces osteoclastogenesis and promotes tumor growth in giant cell tumor of bone

**DOI:** 10.1038/s41419-025-07629-6

**Published:** 2025-09-05

**Authors:** Yunfei He, Dongdong Cheng, Cheng Lian, Yingjie Liu, Wenqian Luo, Yuan Wang, Chengxin Ma, Qiuyao Wu, Pu Tian, Dasa He, Zhenchang Jia, Xianzhe Lv, Xue Zhang, Zhen Pan, Jinxi Lu, Yansen Xiao, Peiyuan Zhang, Yajun Liang, Qingcheng Yang, Guohong Hu

**Affiliations:** 1 CAS Key Laboratory of Tissue Microenvironment and Tumor, Shanghai Institute of Nutrition and Health, University of Chinese Academy of Sciences, Chinese Academy of Sciences, Shanghai, China; 2 Department of Orthopedics, Shanghai Jiao Tong University Affiliated Sixth People’s Hospital, Shanghai, China; 3 Department of General Surgery, Xinzhou District People’s Hospital, Wuhan, China

Correction to: *Cell Death & Disease* 10.1038/s41419-021-04161-1, published online 23 September 2021

In the initially published version of the article, there was an error in Supplementary Fig. S4I. Some of the representative images presented in Fig. S4I were incorrect and have been amended in the PDF version of Supplementary Figures. The misplacement does not affect any conclusion of the paper.

In the article (CDDis 10.1038/s41419-021-04161-1) initially published, some of the representative images (red box) in Fig. S4I were incorrect. The amended version of Fig. S4I is shown below with the correct images (green box).

Corrected Fig. S4:
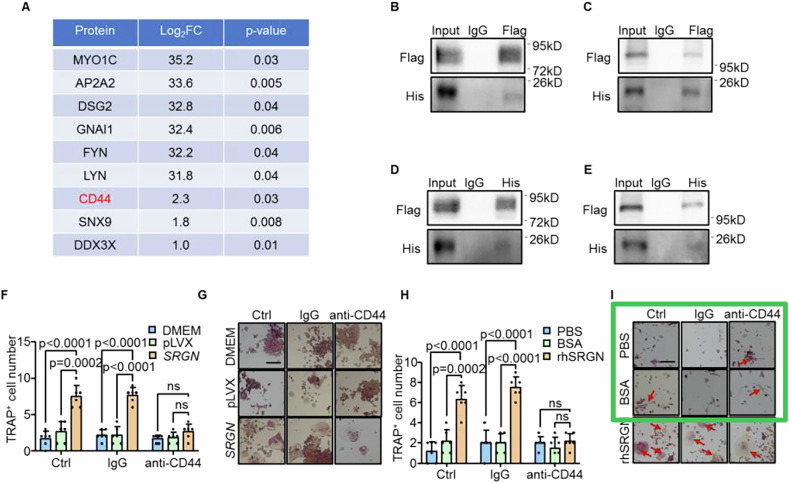


Original Fig. S4:
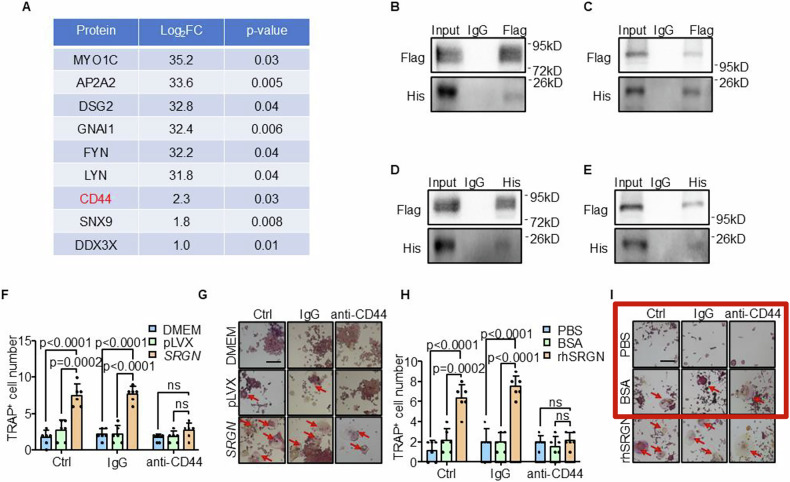


The original article has been corrected.

## Supplementary information

Supplementary_Figures

