# Supplementary material for: Serglycin induces osteoclastogenesis and promotes tumor growth in giant cell tumor of bone
Source: Cell Death Dis. 2021 Sep 23;12(10):868. doi: 10.1038/s41419-021-04161-1 (PMC8460728; doi:10.1038/s41419-021-04161-1)
Supplement: Supplementary file 1 — Supplementary Figures [file 41419_2021_4161_MOESM1_ESM.pdf]

Supplementary Figures

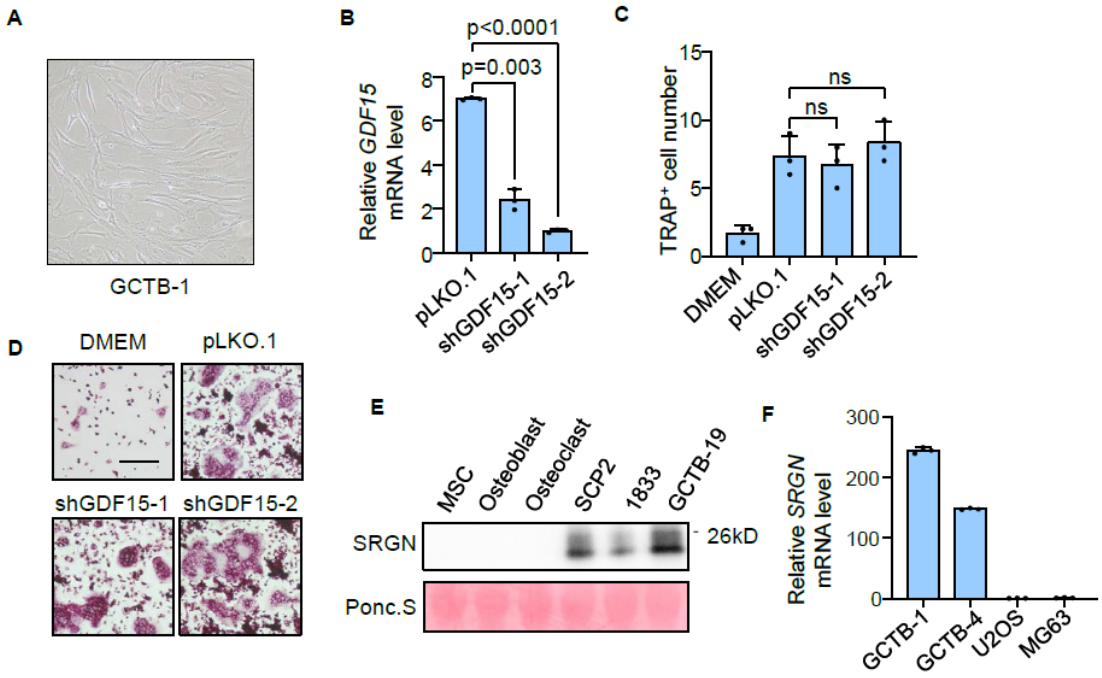

Fig. S1. Analysis of GDF15 and SRGN expression in GCTB.

(**A**) Representative image of the primary stromal cell lines.

(**B**) Quantitative PCR analysis of *GDF15* knockdown in GCTB-1.

(**C-D**) Primary bone marrow osteoclastogenesis assay with the treatment of GCTB-1 conditioned medium (CM). The number of giant mature osteoclasts (**C**) and representative images (**D**) are shown.

(**E**) Secreted SRGN protein in mesenchymal stem cells (MSC), osteoblasts, osteoclasts, breast cancer cell lines (SCP2 and 1833) and GCTB-19.

(**F**) SRGN mRNA levels in GCTB cells with (GCTB-1) or without (GCTB-4) H3.3^{G34W} mutation and osteosarcoma cells.

Scale bar, 100 μm. P values were obtained by two-tailed unpaired *t*-test (**B-C**); ns, not significant. Bar graphs are shown as mean ± s.d.

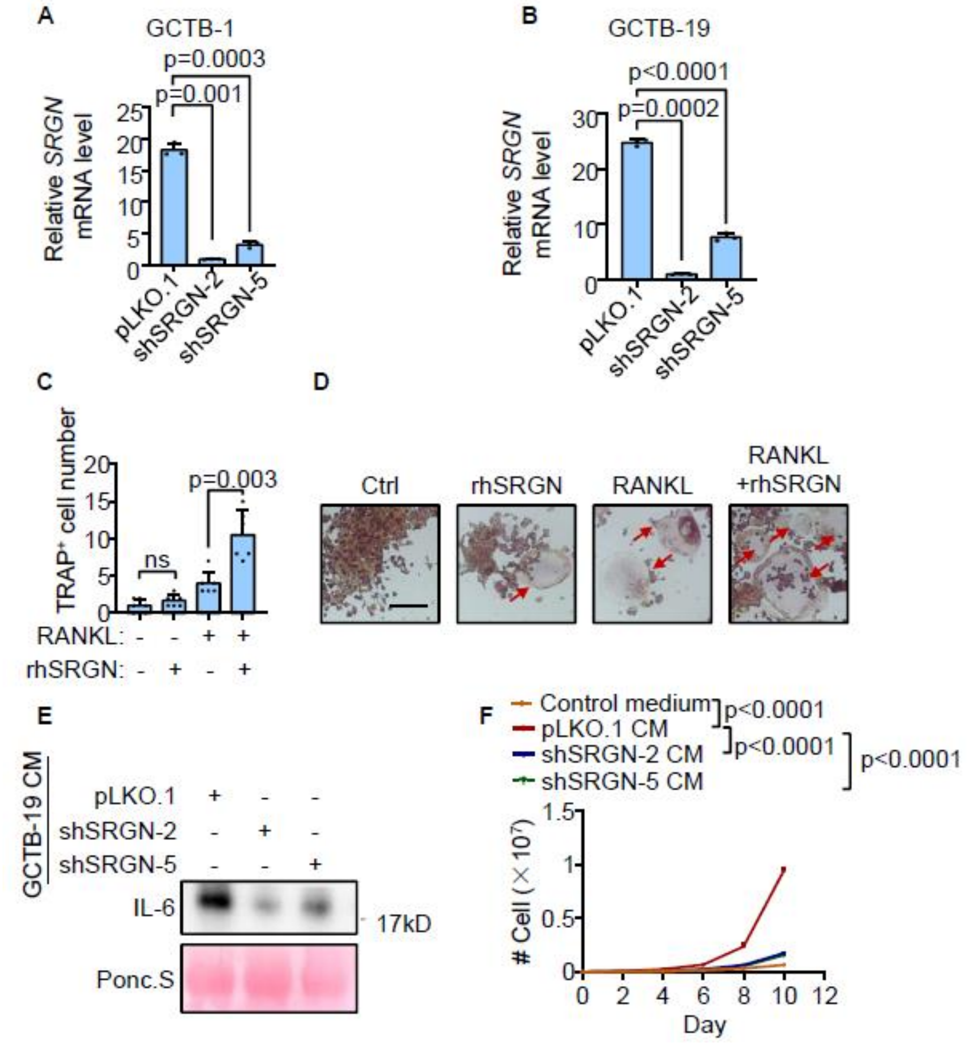

Fig. S2. SRGN promotes osteoclastogenesis and tumor proliferation *in vitro*.

(**A, B**) Validation of *SRGN* knockdown in GCTB-1 (**A**) and GCTB-19 (**B**).

(**C, D**) Osteoclastogenesis (**C**) and representative images (**D**) of RAW264.7 cultured with RANKL and/or recombinant SRGN.

(**E**) Secreted IL-6 level in RAW264.7 induced by conditioned media (CM) from GCTB-19 with or without *SRGN*-knockdown.

(**F**) Growth curve of GCTB-19 cells incubated in control α-MEM medium or CM of RAW264.7 pretreated with GCTB-19 medium with or without *SRGN* knockdown.

Scale bar, 100 μm. *P* values were obtained by two-tailed unpaired *t*-test (**A-C, F**), ns, not significant. Bar graphs are shown as mean ± s.d.

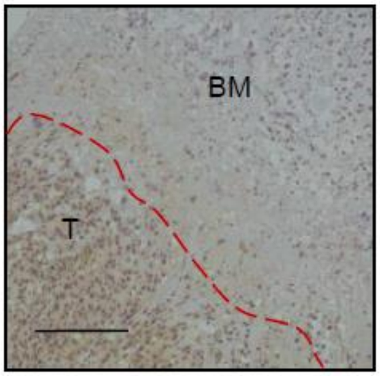

Fig. S3. Immunostaining of SRGN in GCTB tissues of the murine model. T, tumor; BM, bone marrow.

A

Protein	Log_2FC	p-value
MYO1C	35.2	0.03
AP2A2	33.6	0.005
DSG2	32.8	0.04
GNAI1	32.4	0.006
FYN	32.2	0.04
LYN	31.8	0.04
CD44	2.3	0.03
SNX9	1.8	0.008
DDX3X	1.0	0.01

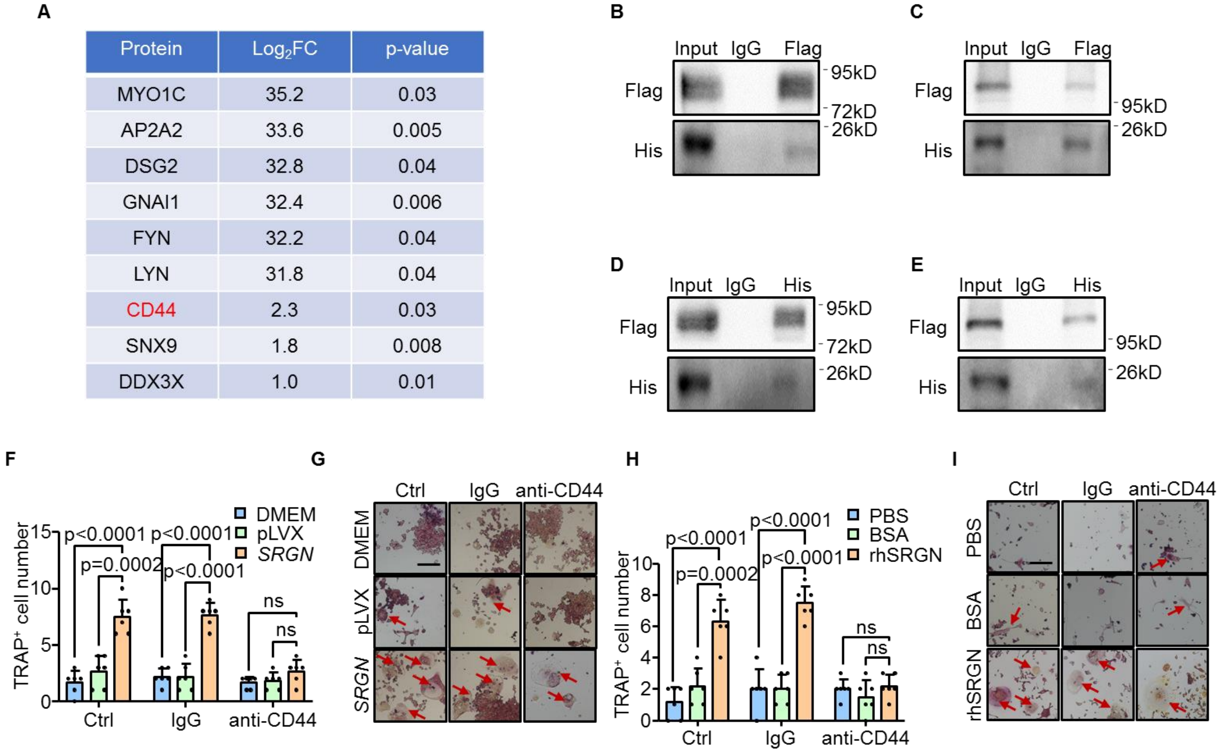

Fig. S4. SRGN functions through its receptor CD44.

(**A**) The list of SRGN-interacting candidate proteins identified with co-IP-Mass spectrum analyses. FC, fold changes of protein counts of anti-SRGN vs. control IgG.

(**B-E**) Co-IP of SRGN and CD44. His-tagged SRGN and Flag-tagged CD44 isoforms, CD44s (**B, D**) and CD44v3-v10 (**C, E**), were overexpressed in 293T cells. Cell lysates were immunoprecipitated with an anti-Flag antibody (**B**, **C**) or an anti-His antibody (**D**, **E**), followed by immunoblotting with anti-Flag and anti-His antibodies.

(**F, G**) Raw264.7 osteoclastogenesis assays with the treatment of control DMEM medium, CM of *SRGN*-overexpressing hFOB1.19 and/or CD44 neutralizing antibody. Shown are the numbers of giant mature osteoclasts (**F**) and representative images (**G**). Arrows point to giant osteoclast cells.

(**H, I**) Raw264.7 osteoclastogenesis assays with the treatment of human recombinant SRGN protein and/or CD44 neutralizing antibody. Shown are the numbers of giant mature osteoclasts (**H**) and representative images (**I**).

Scale bar, 100 μm. P values were obtained by two-tailed unpaired *t*-test (**A, F, H**), ns, not significant. Bar graphs are shown as mean ± s.d.

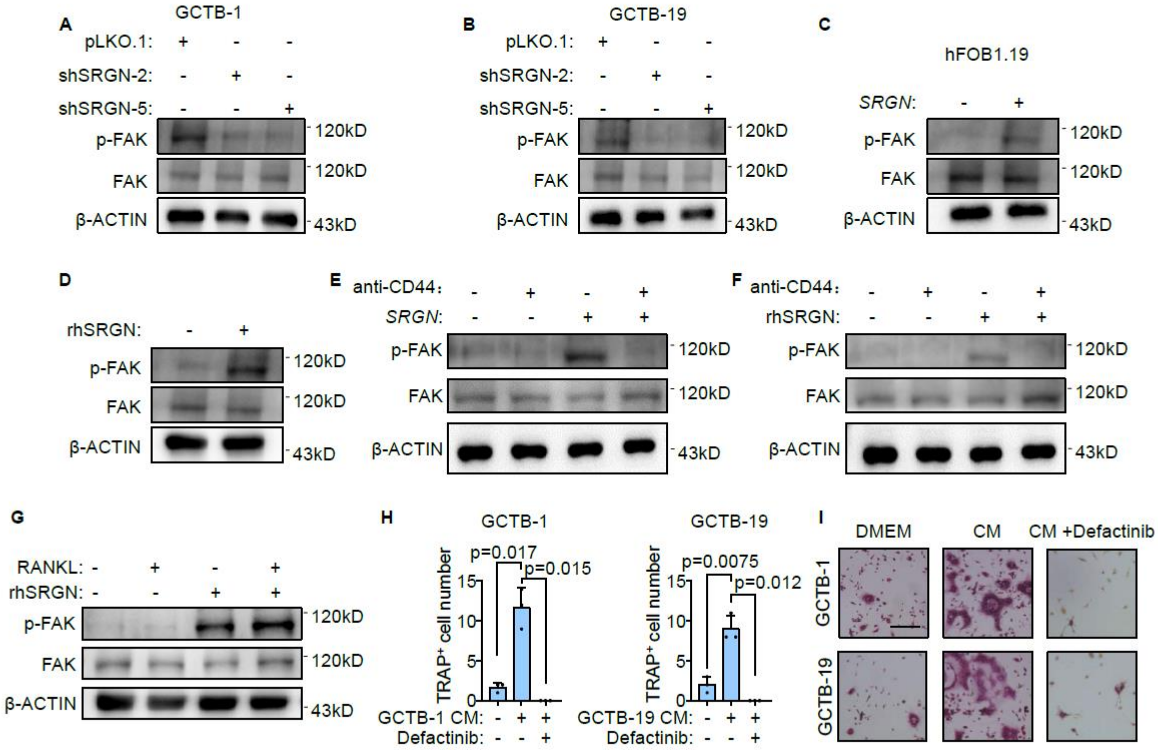

Fig. S5. SRGN activates FAK through CD44.

(**A-D**) FAK phosphorylation in primary bone marrow cells after treatment with CM of GCTB-1 (**A**), GCTB-19 (**B**) and hFOB1.19 (**C**) or human recombinant SRGN protein (**D**).

(**E**) FAK phosphorylation in in primary bone marrow cells after treatment with hFOB1.19 CM and CD44 neutralizing antibody.

(**F**) FAK phosphorylation in in primary bone marrow cells after treatment with human recombinant SRGN protein and CD44 neutralizing antibody.

(**G**) FAK phosphorylation in RAW264.7 cells after treatment with human recombinant SRGN and/or RANKL.

(**H**, **I**) Osteoclast quantification (**H**) and representative images (**I**) in mouse primary bone marrow in CM of GCTB-1 and GCTB-19 with or without Defactinib (2 μM) in osteoclastogenesis assays.

Scale bar, 100 μm. P values were obtained by two-tailed unpaired *t*-test (**G**). Bar graphs are shown as mean ± s.d.
